# Primary Vulval Rhabdoid Tumor in an Adult: A Case Report, Immunohistochemical Profile and Literature Review

**DOI:** 10.1155/2011/162709

**Published:** 2011-09-29

**Authors:** Maria A. Arafah, Muna I. Aljuboury

**Affiliations:** ^1^Department of Pathology, College of Medicine, King Saud University, Riyadh 11421, Saudi Arabia; ^2^Department of Pathology, College of Medicine and King Khalid University Hospital, P.O. Box 2925, Riyadh 11461, Saudi Arabia; ^3^Department of Pathology, Riyadh Armed Forces Hospital, Riyadh 11159, Saudi Arabia

## Abstract

We report a rare case of primary vulval rhabdoid tumor in an adult. The diagnosis was confirmed using the recently emerging INI1/BAF47 immunostain. We also demonstrate the expression of ER and PR hormonal receptors by the tumor cells.

## 1. Introduction

Extrarenal malignant rhabdoid tumors (EMRTs) are rare and highly aggressive neoplasms. Although they are known to occur during infancy and childhood, their occurrence in adults is reported. EMRTs were described in various anatomical sites, and the use of INI1 immunostain has recently emerged as an important adjunct for identifying these tumors. An extensive online search of the literature revealed very few reported cases of EMRT of the vulva [1–10]. We hereby report another case and the first in which the INI1, ER, and PR immunostains were utilized. 

## 2. Case Description

A 25-year-old Saudi female, who is medically free, presented to our emergency department complaining of a mass in the mons pubis. The mass was occasionally tender and had rapidly increased in size in two-week duration. No other complains or masses at other sites were reported. On clinical examination, an ill-defined soft mass was identified in the mons pubis, extending to the left inguinal area with a hyperemic overlying skin. Laboratory tests were within normal limits except for a mild leukocytosis. The clinical impression was of an abscess, and the patient was scheduled for surgery. A local excision of the mass was done, and the specimen was sent to the histopathology department. The patient was discharged on the next day of surgery. 

### 2.1. Gross Findings

Grossly, the specimen consisted of two irregular pieces of soft tissue. Both masses were partially encapsulated and measured 6 × 5 × 2 cm and 6.5 × 6 × 2.5 cm, respectively. The outer surface of both masses was irregular. Serial slicing of the masses revealed a heterogeneous cut surface with pale white and dark tan areas. Random sections from both masses were taken for histopathological examination.

### 2.2. Microscopic Findings

**Figure 1:**
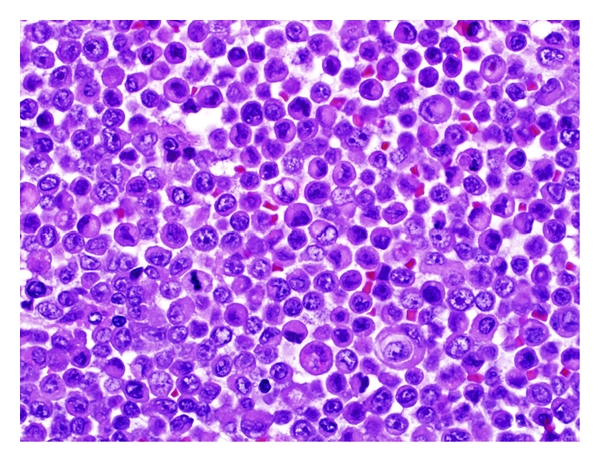
The classical “rhabdoid” morphology of the tumor comprising the cytoplasmic hyaline globules and the eccentric nucleus. Mitotic figures are also seen (H&E stain, magnification power ×400).

H&E slides showed a poorly differentiated neoplasm. The tumor consisted exclusively of cells retaining the classical “rhabdoid” morphology (Figure 1). The cells were discohesive with distinct cell borders, and they were arranged in solid sheets. The amount of cytoplasm varied between scant to abundant. The cytoplasm had a prominent eosinophilic quality, and intracytoplasmic glassy eosinophilic inclusions were seen within the majority of the cells. The nuclei were vesicular, eccentric, and highly pleomorphic with prominent nucleoli. Numerous mitoses were seen (45 mitoses/10 HPF). Occasional multinucleated tumor giant cells were present. There were neither areas of necrosis nor lymphovascular invasion. 

### 2.3. Immunohistochemical Findings

**Figure 2:**
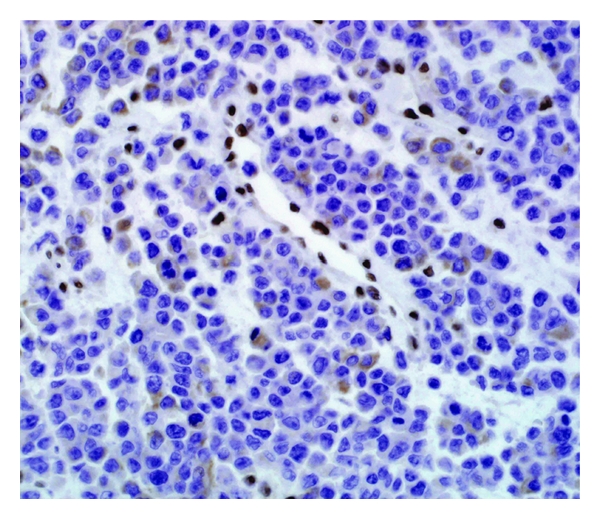
INI1/BAF47 immunostain showing loss of nuclear staining in the rhabdoid cells in comparison to the positive nuclei of the endothelial cells (INI1/BAF47 immunohistochemical stain, magnification power ×200).

**Figure 3:**
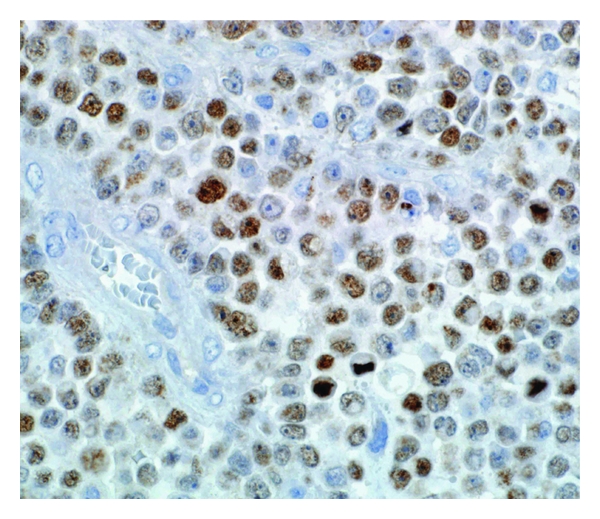
Weak-to-moderate ER nuclear expression of the tumor cells (ER immunohistochemical stain, magnification power ×400).

An extended panel of antibodies was performed on Ventana using the iVIEW DAB detection kit and showed focal cytoplasmic immunoreactivity to vimentin (mouse monoclonal, 1 : 60; Dako) with the characteristic globular cytoplasmic configuration indenting the nucleus. The cytoplasm of tumor cells also coexpressed cytokeratin cocktail (mouse monoclonal, AE1/AE3, 1 : 100; Dako), CK 8 (mouse monoclonal, ready to use; Dako), and CK 19 (mouse monoclonal, 1 : 40; Dako). The tumor cells, in comparison to the endothelial lining of the capillaries, showed loss of INI1 (mouse monoclonal, BAF 47, ready to use; BD Transduction Laboratories, USA) nuclear staining (Figure 2). Anti-Ki-67 (mouse monoclonal, 1 : 50; Dako) showed a high proliferation index reaching up to 80% of tumor cells. Tumor cells have showed nuclear positivity to ER (mouse monoclonal, 1 : 20; Dako) (weak to moderate in 90%) (Figure 3) and have also showed nuclear positivity to PR (mouse monoclonal, 1 : 100; Dako) (strong in 20%). All other immunohistochemical (IHC) stains were negative including CD34, EMA, CD99, CD117, CD45, CD30, CD138, bcl-6, bcl-2, CK7, CK20, GCDFP, muscular differentiation markers (Desmin, SMA, Myoglobin), neuroendocrine markers (Synaptophysin, Chromogranin A, CD56), and melanoma markers (S100, HMB-45). 

A diagnosis of malignant rhabdoid tumor of the vulva was established. The patient was recalled once the histopathology report was signed out. A computed tomography scan (CT) of the head, chest, abdomen, and pelvis was performed and revealed no other tumor masses. Unfortunately, the patient failed to show for followup.

## 3. Discussion

We report a case of EMRT of the vulva in a 25-year-old female. The age of the patient, the site of the tumor, the clinical presentation, and the microscopic findings in our case were compatible with the 12 previously  reported cases (Table 1) [1–10]. The IHC profile varied between cases, and the only universal finding was immunoreactivity to vimentin. Recently, INI1 immunostain has emerged as an adjunct for identifying rhabdoid tumors and atypical rhabdoid/teratoid tumors (AT/RTs). INI1, encoded by the INI1/hSNF5/SMARCB1/BAF47 locus at 22q11.2, is a member of the SWI/SNF chromatin-remodeling complex, and it is normally expressed in all tissues [11, 12]. The biallelic inactivation of this gene is characteristic to rhabdoid tumors of the kidney, EMRTs, and AT/RTs [11, 12]. We have confirmed our diagnosis using the INI1/BAF47 antibody which showed universal loss of nuclear INI1 protein expression in all tumor cells. We have also studied the hormonal status of this tumor and found it to express both ER and PR receptors. This finding is compatible with its primary location in the vulva and has been previously reported in other vulval tumors [13].

Malignant rhabdoid tumor was originally described in the kidney as a variant of Wilms' tumor. In 1982, the first extrarenal malignant rhabdoid tumor was reported, and since then this entity was recognized at various anatomical sites including soft tissues, liver, prostate, thymus, skin, pelvis, heart, and central nervous system [14, 15]. These tumors have a predilection to the pediatric age group though cases in adults were repeatedly recognized. The common microscopic feature is the “rhabdoid” cell of which the entire tumor is composed. These tumors usually coexpress vimentin and cytokeratin, and they do not retain the nuclear expression of INI1 protein. The prognosis of ERMT is generally poor, and their course tends to be aggressive. Although surgery is the first line of management, there is no consensus that exists regarding adjuvant therapies. Chemotherapy and radiotherapy did not show to be effective in controlling the recurrent or metastatic disease in all reported cases [1–15]. 

In summary, vulval EMRTs are rare and aggressive neoplasms and should be considered in the differential diagnosis of any high-grade undifferentiated tumor at this site. Given that IHC is much more widely available than molecular tests, INI1/BAF47 immunostain represents the most useful, up to date ancillary technique to confirm this diagnosis. Although these tumors express ER and PR receptors, their response to antihormonal therapy is a remote possibility [13].

## Figures and Tables

**Table 1:** Characteristics of the previously reported cases of EMRT of the vulva [1–10].

Author, year	Age at diagnosis (years)	Site	Primary treatment	Reported survival (months)
	19	R LMJ		38 (alive)
Perrone et al., 1989^§^ [1]	30	R LMJ	Surgery	8 (dead)
	31	L vulva		138 (dead)
Matias et al., 1990 [2]	49	L LMJ	Surgery + chemotherapy	9 (dead)
Lupi et al., 1996 [3]	39	L LMJ	Surgery + chemotherapy + radiation	4 (dead)
Igarashi et al., 1998 [4]	39	L LMJ	Surgery + chemotherapy	8 (alive)
Sert et al., 1999 [5]	44	R LMJ	Surgery + chemotherapy + radiation	8 (dead)
Brand and Covert 2001 [6]	40	Mons pubis	Surgery + chemotherapy + radiation	61 (alive)
Haidopoulos et al., 2002^¥^ [7]	—	Clitoris	—	—
Tzilinis et al., 2002 [8]	63	L LMJ	Surgery + radiation	30 (alive)
Argenta et al., 2007^€^ [9]	35	R vulva	Surgery + radiation	40 (alive)
Narendra et al., 2010 [10]	50	R LMJ and mons pubis	Surgery + radiation	30 (alive)
Current case	25	Mons Pubis, extending to L inguinal area	Surgery	Lost followup

R: right. L: left. ^§^3 cases were reported. ^¥^No full text is available in PubMed. ^€^The diagnosis given was EMRT/proximal epithelioid sarcoma.
